# A mobile hospice nurse teaching team’s experience: training care workers in spiritual and existential care for the dying - a qualitative study

**DOI:** 10.1186/s12904-015-0042-y

**Published:** 2015-09-18

**Authors:** Kirsten Tornøe, Lars Johan Danbolt, Kari Kvigne, Venke Sørlie

**Affiliations:** 1 Lovisenberg Diaconal University College, Lovisenberg gt.15B, 0456 Oslo, Norway; 2 MF, Norwegian School of Theology, Gydas vei 4, Majorstuen, P.O. Box 5144, 0302 Oslo, Norway; 3 Religionspsykologisk Senter (Center for the Psychology of Religion), Innlandet Hospital, P.O. Box 68, 2312 Ottestad, Norway; 4 Department of Nursing, Faculty of Public Health, Hedmark University College, P.O. Box 400, 2418 Elverum, Norway; 5 Department of Nursing, Nesna University College, Nesna, Norway

**Keywords:** Palliative spiritual care, Primary health care, Staff training, Phenomenological hermeneutical

## Abstract

**Background:**

Nursing home and home care nursing staff must increasingly deal with palliative care challenges, due to cost cutting in specialized health care. Research indicates that a significant number of dying patients long for adequate spiritual and existential care. Several studies show that this is often a source of *anxiety* for care workers. Teaching care workers to alleviate dying patients’ spiritual and existential suffering is therefore important. The aim of this study is to illuminate a pioneering Norwegian *mobile hospice nurse teaching team’s* experience with teaching and training care workers in spiritual and existential care for the dying in nursing homes and home care settings.

**Methods:**

The team of expert hospice nurses participated in a focus group interview. Data were analyzed using a phenomenological hermeneutical method.

**Results:**

The *mobile teaching team* taught care workers to identify spiritual and existential suffering, initiate existential and spiritual conversations and convey consolation through active presencing and silence. The team members transferred their personal spiritual and existential care knowledge through situated “bedside teaching” and reflective dialogues. “The *mobile teaching team* perceived that the care workers benefitted from the situated teaching because they observed that care workers became more courageous in addressing dying patients’ spiritual and existential suffering.

**Discussion:**

Educational research supports these results. Studies show that efficient workplace teaching schemes allowexpert practitioners to teach staff to integrate several different knowledge forms and skills, applying a holisticknowledge approach. One of the features of workplace learning is that expert nurses are able to guide novices through the complexities of practice. Situated learning is therefore central for becoming proficient.

**Conclusions:**

Situated bedside teaching provided by expert mobile hospice nurses may be an efficient way to develop care workers’ courage and competency to provide spiritual and existential end-of-life-care. Further research is recommended on the use of mobile expert nurse teaching teams to improve nursing competency in the primary health care sector.

## Background

Nursing homes and home care nursing must provide more end-of-life-care, due to the international trend of downsizing hospital units and cutting health care costs in secondary health care [1, 2]. Henceforth, care workers in primary health care will increasingly encounter dying patients’ spiritual and existential suffering.

In 2010, managers in a leading hospice collaborated with primary health care administrators in a major Norwegian city, to create a pioneering *“mobile hospice nurse spiritual and existential care teaching team”.* Their aim was to teach and train care workers in spiritual and existential care for the dying in nursing homes and home care nursing. Nursing home and home care managers requested *the mobile teaching team’s* services to provide on-the-job-support and supervision for care workers who felt anxious and uncertain about engaging in spiritual and existential care for dying patients. *The mobile teaching* team supervised registered nurses as well as state enrolled nurses and unregulated nursing assistants, because care workers could be anxious and uncertain about spiritual and existential end-of-life-care, regardless of their professional status. The different categories of nursing staff will therefore be referred to as care workers in this paper.

Dying patients frequently experience severe spiritual and existential distress [3]. Bruce et al. [4] described existential suffering as a condition where morbid suffering may include concerns related to hopelessness, futility, meaninglessness, disappointment, remorse, death anxiety and a disruption of personal identity. Research indicates that a significant number of dying patients long for adequate spiritual and/or existential care and counselling [5, 6]. Patients with advanced illnesses report that their medical caregivers infrequently provide spiritual care [7]. According to Udo [8] several studies show that many patients are dissatisfied with the emotional and existential support they are given.

According to Puchalski et al. [9] most care settings fail to provide optimal spiritual care to those with serious illness and those at the end of life. Several studies show that registered nurses and other care workers often are inadequately prepared and feel anxious and uncertain about providing spiritual and existential care for the dying [8, 10–14]. This may lead to unmet spiritual and existential needs, resulting in increased patient suffering [7]. Henceforth, there is a widespread need for training in all aspects of spiritual and existential end-of-life-care [7, 15].

Several nurse educators have grappled with the complex challenges of developing a curriculum and strategies to teach spiritual care [16–19]. However, given the abstract nature of spirituality, teaching spiritual care is more complex than teaching concrete dimensions of care [20]. Providing spiritual care is a process with no fixed answers [8]. Care workers might therefore benefit from receiving spiritual and existential end-of-life-care training from expert nurse practitioners in the workplace [21–25].

In 2014, Pesut et al. [26] conducted a scoping review to summarize the available evidence concerning palliative care education for nurses and other nursing care providers. None of the references in their review explicitly mentioned training care workers in spiritual and existential end-of-life-care. This suggests that there exists a gap in the literature concerning this topic.

As there seems to be no single agreed definition of spiritual care in the research literature, the term is open to interpretation [10, 13, 27–29]. This study has therefore adopted a pragmatic and functionalist epistemological point of departure since it is targeted at the practical implications of *the mobile teaching team’s* experience, rather than the ontological questions related to the conceptual framework of spiritual care.

### Aim

The aim of this study is to illuminate a pioneering Norwegian *mobile hospice nurse teaching team’s* experience with teaching and training care workers in spiritual and existential care for the dying in nursing homes and home care settings.

## Methods

### Design

The study was conducted using a qualitative phenomenological hermeneutical approach influenced by Ricoeur’s philosophy [30], and is suitable for illuminating lived experience [31–33].

### Participants

As Norway’s first and only *mobile spiritual and existential care teaching* team in end-of-life care, the team was invited to participate in a focus group interview. The team members were expert hospice nurses from a leading hospice in a major Norwegian city. They had several years experience as clinical supervisors in end-of-life care, and were between fifty-five and sixty-one years old, having from five to fifteen years experience with hospice nursing. The team members represented a broad range of nursing expertise, holding advanced nursing degrees in a variety of fields, such as oncology nursing, palliative care, intensive care, psychiatric care, substance abuse nursing and clinical supervision.

### Data collection

The focus group interview was chosen because it is an efficient way to obtain data from informants who work together daily: «…*colleagues can relate to each other’s comments to incidents in their daily shared lives. They may even challenge each other on contradictions between what they profess to believe and how they actually behave*» [34 p.300]. The interview was conducted in the Hospice meeting room by the first author. The fourth author functioned as secretary, taking field notes to comment on situational aspects, language and interaction [35]. The interview lasted 80 min.

The interview strategy was designed as a narrative approach, with one open-ended question, followed by clarifying questions when necessary: *What are your experiences with teaching and supervising care workers in existential and spiritual care for the dying*?

This choice was based on the presupposition, that the interviewees’ perspectives are best revealed in narratives where they use their spontaneous language in the narration of events [36, 37]. We did not introduce any definitions of spiritual and existential care at the commencement of the interview, in order to allow the participants to talk freely about what they considered as spiritual and existential care. The aim of the interview was to obtain as many rich narratives as possible, by minimally interrupting the participants’ narrative flow and reflection. In order to obtain the meaning of the participants’ narratives [38] the researchers followed up on the themes they focused on during the interview. This was done by tying questions and comments to their narratives and repeating their words whenever possible [39]. The interview was audiotaped and transcribed verbatim by the first author.

### Data analysis

Data were analyzed using Lindseth & Norberg’s [31] phenomenological hermeneutical method for researching lived experience, where each interview is looked upon as a text. The interpretation implies a dialectic movement between the text as a whole and parts of the text, and consists of three steps: The first step was a *naive reading* to grasp an overall impression of the text. This gave access to the participants’ lived experience with teaching spiritual and existential care. Keeping an open mind, the transcribed interview was reread many times. The analysis moves towards a phenomenological world, allowing the researchers to be touched by the narratives. The naïve understanding of the text reveals the direction for the structural analyses [30, 31, 40, 41]. The naïve reading was discussed between the researchers and further guided the structural analysis. The structural analysis was the second step. The text was divided into meaning units. These were condensed into themes. The objective of the structural analysis was to explain what the text was saying. The themes were compared with the naïve reading to validate the structural analysis. We found the themes to be consistent with the naïve reading. This strengthened the validity of the analysis. The themes are presented in the Results section. Finally, a *critical comprehension* was developed. The text was read as a whole, taking into account the authors’ preunderstanding, naive reading, structural analysis, previous research and relevant theory [31, 33, 42]. The critical comprehension is presented in the Discussion section.

### Rigour

The interview provided a large amount of in-depth information about the meaning of the team members’ lived experience. A text may have more than one possible interpretation and the interpretation presented here should be looked upon as one possible, but not the only way of understanding their experiences [31, 33]. To ensure rigour, the authors performed individual structural analyses. The interpretations were compared to strengthen the validity of analysis. All authors critically reviewed and discussed the interpretation of the results.

### Methodological considerations

Sample size determination in focus group studies is a complex issue that is debated in the literature. The textbooks usually recommend from two to five groups per category of participants. There is however, no consensus on sample size. The actual size and group composition depends on the research question and the characteristics of the participants. The main question is whether the data are sufficiently diverse and rigorous to answer the research question [43–45]. It is essential that potential participants are selected on the basis of their ability to provide insight into and information about the research topic and that they are able to articulate their perspective on relevant issues [46].

Since *the mobile teaching* team consisted of three members, the number of key informants limited itself. The team members were selected because of their unique experience and expertise as the first and only mobile spiritual and existential care teaching team in Norway. The fourth author was familiar with the mobile teaching team from previous research projects at the hospice, and knew that they were able and willing to participate in research. The team members knew each other well because they worked together on a daily basis. We therefore assumed that one eighty minute focus group interview would produce a sufficient amount of in-depth data. The interview transcript, yielding thirty pages of thick descriptions, confirmed this. Drawing on Sandelowski, Carlsen and Glenton [43] point out that too many groups can lower the quality of focus group studies. The more pages of transcribed interviews, the less depth and richness the authors will be able to extract from the material. We argue in line with Pope et al. [47] that since qualitative studies are not designed to be representative in terms of statistical generability, they may gain little from expanding sample size except a more cumbersome data set.

Lindseth and Norberg’s [31] Ricoeur inspired phenomenological hermeneutical interpretation method was chosen to analyze the data since it is designed to disclose the meaning of lived experience in narrative interviews. We are however well aware that combining a phenomenological hermeneutical interpretation method with focus group interviews is debated in the research literature [48, 49].

We will justify our design choices in the following: Nurse researchers highlighting the incompatibility of focus groups and phenomenology, claim that phenomenology’s emphasis on individual, lived experience is inconsistent with group approaches [48, 49]. This is contested by Morgan [50], who claims that focus group discussions depend on both the individuals that make up the group and the dynamics of the group as a whole. *“Although the influence of the group on the individual participants is undeniable, this is a far cry from demonstrating that the group should be the unit of analysis in focus group research”* [50 p.60]. We argue that individual perspectives can still be preserved in a group context. In a small focus group participants can tell their narratives as self-contained stories. Other group members can then add valuable perspectives as the story unfolds, probing for more information and adding their own insights related to shared meanings. Henceforth, it can be argued that a group approach does not exclude individual perspectives – rather, it includes them [51]. Our focus group consisted of only three participants, making it possible to give sufficient time for each individual to share their experiences. Care was taken to moderate the group so that all participants were heard. The participants reflected openly about their experiences, confirming and reinforcing each other’s views.

The focus group can help researchers to bracket prejudices because group members may challenge their assumptions [51]. Group interaction gives access to the participants’ common sense conceptions and every day explanations and experiences [51]. The focus group stimulates discussion, opens up new perspectives, encourages exchange and enriches and complements [51]. The focus group discussion is recorded and transcribed into a text. According to Ricoeur’s interpretation theory [30, 52] a written text breaks away from its author’s intention, its original, social and cultural setting, and from its original audience as well [53]. *“The written text frees us from the visibility and limitations of situations by opening up a world for us, that is, new dimensions of our being- in –the- world”* [52 p. 145]. According to Ricoeur [30] one person’s experience cannot directly become another person’s experience. Nevertheless “something” is transferred from one sphere of life to another. Ricouer [30] claims that this “something” is not the experience as experienced, but its meaning. *“The experience as experienced, as lived, remains private, but its’ sense, its' meaning, becomes public. In this way, communication is the overcoming of the radical non-communicability of the lived experience as lived* [30 p.16]. Therefore, the aim of the phenomenological hermeneutical analysis is to interpret the meaning of the interview text, rather than the experience of individual participants. According to Lindseth and Norberg [31] the phenomenological hermeneutical researcher *“does not want to seize these experiences as something “factual” as psychic, social or historical events that need explanation. As phenomenologists we want to focus on the understandable meaning of these experiences*”[31 p. 146].

As this is a qualitative study, it is not reasonable to discuss the concepts of validity, reliability and generalizability in their traditional senses. The number of informants in qualitative research projects is not sufficient to allow for generalized conclusions. However, they do insure strength and representativity in relation to transferability, as they permit an in-depth insight into the phenomena under study. Qualitative projects can therefore be stated to show a high content validity. This means that there is a high degree of detail in the data [54]. According to Mishler [36] and Kvale and Brinkmann [55], three to five informants are sufficient to achieve a high content validity.

### Ethical considerations

The study was conducted according to the Helsinki declaration. Approval was obtained from the Norwegian Social Science Data Service (NSD), project number 29973 and participants gave their written consent.

## Results

Three themes emerged through the structural analysis: *Fear and Uncertainty, Bedside teaching, Courage and Competency*. In the text, citations are used to illustrate the results.

### Fear and uncertainty

According to *the mobile teaching team*, care workers often expressed that they felt reluctant to address dying patients’ existential and spiritual suffering. *The mobile teaching team* said that care workers could be quite afraid of talking with patients about their existential and spiritual concerns. According to the team members, care workers could hesitate to ask how patients “*really felt*”, because they were afraid of not being able to answer the patients’ spiritual and/or existential questions:

Nurse 1: *“In my experience a lot of care workers are scared of death. They don’t dare to talk with the patients about dying, because they are so uncertain about what to say.”*

Nurse 2: “*You know, when you’re “out there” some of the care workers want us to deal with the patients’ spiritual needs for them. But my goal is to build up their courage and confidence so they can do it themselves. I try to show them how to create natural openings to talk with patients about these things”.*

Nurse 3: “*Yes, but there is also a lot of healing in sharing the silence. So I try to show them how important that is. But its really hard for some, because they are so scared of being with the dying that they try to avoid staying in the room with them.”*

The *mobile teaching team* tried to help the care workers to understand that they could convey consolation, just by active listening and sharing moments of silence with the patients. Care workers were encouraged to do this. However, *the mobile teaching team* often experienced that *“just being there”*, could often be too challenging: *“Many of them are afraid of silence and just being with the patient in the room of death. They need to be able to use themselves as an instrument, becoming more courageous, and daring to investigate how the patient experiences his situation.”* In the team members’ opinion, care workers’ fear of exposing themselves to patients’ existential and spiritual suffering stemmed from personal insecurity as well as insufficient communication and listening skills.

### Bedside teaching

*The mobile teaching team* taught care workers to identify patients’ spiritual and existential suffering, initiate existential and spiritual conversations and convey consolation through silent presencing and active listening. They transferred their personal spiritual and existential care knowledge by participating actively in patient care together with the care workers. *The mobile teaching team* gave care workers supervision, and feedback directly related to these situations. They called this “bedside teaching”. Bedside teaching could take place during many kinds of patient encounters, such as giving physical care, conducting nursing procedures, or just taking part in conversations with patients.

Inspired by their holistic hospice values, *the mobile teaching* team strove to teach care workers to *“work from the heart”,* emphasizing the relational aspect of care and each patient’s uniqueness. Using their practical understanding and experience, the team members taught care workers to integrate the physical, social, psychological and spiritual dimensions into a holistic approach to spiritual and existential care: *“…- You can’t talk about spiritual or existential warmth when the patient lies there in spasms of pain. If the pain isn’t relieved, − forget it!”*

The *mobile teaching team* was frequently summoned to help care workers in nursing homes and homecare to communicate more skillfully in “*the difficult conversations around death and dying-“where they didn’t know what to say”.* By acting as role models in these situations, *the mobile teaching team* showed care workers how they could encourage patients to vent their spiritual and existential distress: *“Sometimes they need to hear the kind of questions I ask and see how I relate to the patient”. “Did you see what I did? “Did you notice how straightforward I was?” Did you notice how the patient reacted?*

The team members also demonstrated how they used natural opportunities during physical care to assess spiritual and existential needs and integrate appropriate spiritual and existential care. Simply asking, “*How are you?”* could be enough to “*open the door to meaningful and safe dialogues with patients about their thoughts and feelings*”. *The mobile teaching team* taught care workers to listen actively and pay attention to the patient’s facial expressions and body language.

In addition to role modeling, they also supervised and encouraged care workers to gradually conduct the patient conversations independently. Care workers were encouraged to enter the room with the simple question*“What do you need from me today?”* The team members provided support by accompanying care workers in the conversations, albeit staying in the background as much as possible. In *the mobile teaching team’s* opinion some care workers underestimated themselves: “*Many just need a “little push” and encouragement to talk with the patient alone, using me as a conversation partner to help them reflect on how they handled the situation”.*

In *the mobile teaching team’s* early days, the team members had tended to *“take over too much”,* eagerly demonstrating *“how to do it*”. However, as the team members gained experience, they gradually learned to *“walk in step with the care workers”, and communicate on their “wave length.”* Acting less as instructors and more as supervisors, *the mobile teaching team* encouraged *“learning by doing”* to help care workers develop their courage and competency to alleviate spiritual and existential suffering.

*The mobile teaching team* stressed that critical reflection was an important part of “*learning by doing”*, and initiated reflective dialogues with the care workers about their spiritual and existential care challenges, before and after patient encounters. *The mobile teaching team* experienced that these conversations often evolved around the challenges of: “*finding out how the patient really is, talking about the painful and difficult things related to death and suffering, providing hope in hopeless situations, becoming aware of and talking with patients about their religious or spiritual concerns, collaborating with the chaplain, how they could endure in their work, being present in the “room of death” and in the patient’s suffering, daring to sit down and be quiet together with the patient, being a fellow human being and using oneself as an instrument”.*

### Courage and competency

The *mobile teaching team* reflected on their experiences with teaching and training care workers in spiritual and existential end-of-life-care through situated bedside teaching. Drawing on care worker feedback and their own observations, they considered that situated “bed-side teaching” had proven to be an important tool to develop care workers’ courage and competency to provide spiritual and existential care for the dying:

*“When I have accompanied the same care workers to the same patients several times, I’ve noticed that they have gradually become braver, because they actually dare to ask their patients some of the difficult questions.”*

*The mobile teaching team* observed that care workers became more involved and willing to expose themselves to their patients’ spiritual and existential suffering. They thought that this indicated that the care workers had become more courageous: *“I see that they dare to involve themselves more in these situations, exposing their vulnerability. I see that they have become braver.”*

*The mobile teaching team* said they experienced that many care workers became more frustrated because they saw the patients’ needs more clearly after receiving situated bedside teaching. Based on their observations, *the mobile teaching team* thought that care workers had become more engaged in alleviating their patients’ suffering: *“You see, when they learn from us, they become frustrated, saying things like:” But we don’t have enough room! We don’t have the resources we need”. Then I answer: −Well, have you used your knowledge to ask for more time and resources? - Have you documented that the patient has existential or spiritual concerns, and needs someone to talk to? - And then they have gone and done that. So you see, I think it’s rewarding, when they vent their frustration during supervision, because I think this shows that they have become more involved. And then I’ll ask them to reflect: “What do you think you can do? So I dare say that their competency has improved.”*

## Discussion

In this study the *mobile teaching team* narrated about their experiences with teaching and training care workers in spiritual and existential care for the dying. To develop a critical comprehension (the last step in the analysis), the text was read as a whole, taking into account the authors’ preunderstanding, naïve reading, structural analysis, previous research and relevant theory.

*The mobile teaching team* experienced that care workers’ main obstacle to engage in spiritual and existential care was their fear and uncertainty of facing dying patients’ suffering. It seems reasonable to believe that the care workers’ fear and uncertainty was driven by their own feelings of anxiety related to the unpredictable reality of illness, suffering and dying [56]. According to Popovic [57], anxiety can be seen as an affective expression of our awareness of uncertainty. Our results are supported by Bruce et al’s [4] study. They found that caring for patients with irresolvable suffering exposed the care workers to their own anxiety of experiencing fear, pain and suffering. This made them vulnerable to their latent fear and anxiety of death and dying:*” The struggle in someone else’s life opens up fears and anxieties about the transient nature of our own lives on earth…. Maybe not just the fact that we will die, but the fact that we may suffer or face fear and pain”*[4 p.3]. This anxiety is understandable in light of Yalom’s [58] groundbreaking work on existential psychiatry. According to Yalom [58], death is a primordial source of anxiety: “*Occasionally some jolting experience in life tears a rent in the curtain of defenses and permits raw death anxiety to erupt into consciousness. Rapidly however, the unconscious ego repairs the tears and conceals once again the nature of the anxiety*” [58 p.44]. This suggests that care workers’ reluctance to be with patients *“in the room of death”* was generated by a need to distance themselves (in Yalom’s words), to prevent *“raw death anxiety from erupting into consciousness”.*

This seems reasonable considering that death is a taboo subject in our society [59]. Philippe Aries points out that western culture marginalizes death and dying [60, 61]. Western society’s view that nature exists for us to use and control makes it more difficult for people to experience and accept severe illness and functional decline as a part of life [62]. Due to increased institutionalization and medicalization of the suffering and dying, fewer people have experiences with death and therefore fear it more [62, 63]. Western medicines’ advances and preventive care practices have spawned unrealistic expectations of mastering suffering and dying. Moreover, the unaesthetic aspects of aging, suffering, and dying stand in stark contrast to western society’s beauty and body worshipping [63]. As a consequence, we live in a society where many people are psychologically unprepared and fearful of facing the existential uncertainties that accompany aging, suffering and dying [62, 64].

Nevertheless, encountering work related existential uncertainty is a reality in the lives of care workers involved in end-of-life-care. According to Penrod [65] experiencing uncertainty is a discomforting feeling that can be mediated by feelings of confidence and control. However, existential uncertainty is a fact of life that cannot be solved or controlled “*by becoming more certain*” [33 p. 44]. Care workers must therefore learn to live with existential uncertainty in order to endure bearing witness to dying patients’ spiritual and existential suffering.

Vaismoradi et al. [66] point out that uncertainty is an inescapable and omnipresent fact of decision-making in nursing because people experience complex life events surrounding health. In health care as in other areas of human activity, judgment and decision tasks are uncertain. Professionals are therefore legitimately unsure and uncertain about what is the right or best thing to do. Nevertheless, care workers must make choices in almost every patient encounter [4]. Courage is therefore demanded, because a window is opened to the unknown [67]. In this light, alleviating dying patients’ spiritual and existential suffering can be understood as acts of courage [68].

The mobile teaching team considered that bedside teaching was an essential means to enable the care workers to become more courageous “*to be with patients in the room of death”.* According to them, bedside supervision had strengthened the care workers’ courage and skills to alleviate their patients’ spiritual and existential suffering, because they had observed that care workers gradually became more daring and willing to involve themselves in the patients’ situation and to talk with them about their concerns related to suffering and dying.

Ohlén [69] points out that suffering patients can receive both comfort and strength, and thereby courage to face life, from fellow human beings who are present, and who show, through their actions, that they will be by his or her side and try to share the hard times.

According to Aristotle, human action is a practical skill. From this perspective, knowledge can be understood as *episteme* (theoretical knowledge), *techne* (hands on skills) and *phronesis* (the personal ability to take action in a wise and prudent manner) [69–71]. Ohlén [69] states that all three knowledge forms are each other’s prerequisites. Any one of them alone is not enough. The ability to act prudently and wisely *(phronesis)* presumes theoretical knowledge *(episteme)* about suffering and alleviating suffering as well as hands-on nursing skills to alleviate suffering *(techne)*. However, Ohlén [69] emphasizes that neither theoretical knowledge nor hands-on skills can alleviate suffering unless they are applied with sensitive judgment and prudence *(phronesis)*. In the setting of caring for the very ill and dying *“phronesis”* or *practical wisdom* understood as the care workers’ ability to meet the suffering person and to act with sensitivity and openness becomes important [69]. This is in line with our results. For *the mobile teaching team*, teaching spiritual and existential care involved more than conveying theoretical principles *(episteme)* and practical nursing skills *(techne). The mobile teaching team* placed great importance on teaching care workers to *“work from the heart”,* emphasizing the relational aspects of care and each patient’s uniqueness. To alleviate spiritual and existential suffering, care workers must learn to be responsive and open in encounters with dying patients and their families [69]. In acting sensitively and openly, there is an implicit acceptance of the moral responsibility for the other person as a basis for the relationship. This kind of sensitivity and openness is characterized by the care workers’ actions that arise from proper and appropriate intentions [69, 72].

According to Eraut [23] efficient work place teaching schemes allow practitioners to teach staff to integrate several different forms of knowledge and skills, applying a holistic approach to knowledge. By sharing their practical wisdom and experience with the care workers, the team members demonstrated how to integrate the physical, psychological, existential and spiritual dimensions in a holistic and sensitive approach to spiritual and existential end- of- life care.

One of the features of learning in a practice context is that experts are able to guide novices through the complexities of practice [73]. According to Lave & Wenger [74] situated learning is central for becoming proficient. Learning in practice is a matter of acculturation, of joining a community of practice, rather than the application of decontextualized skills and principles. *The mobile teaching team’s* bedside teaching approach provided the care workers with situated learning experiences in spiritual and existential care. Brown et al. [75] describe the way novices learn from experts as a “*Cognitive apprenticeship*”. One of the defining characteristics of cognitive apprenticeship is that experts make their situational and tacit knowledge explicit as they coach the learner. Much clinical knowhow can only be demonstrated as the particular situation arises. The variety and exceptions in actual clinical practice elude textbook descriptions but gradually yield to the experienced nurse’s fund of past similar and dissimilar situations. It is this demonstration that is so essential to the novice [76]. According to Brown et al. [75] experts make use of the following strategies to support novices as they develop their competence. These include *modeling, scaffolding, fading, articulation, reflection and exploration*. All of these strategies were found in *the mobile teaching team’s* bedside teaching practice. *Modeling* involved showing care workers how they communicated with the patients: *“Sometimes they need****to hear*** *the kind of questions I ask* ***and see*** *how I relate to the patient”*. Afterwards, the team members would draw attention to key features from the conversation: *“Did you notice how straightforward I was?” Did you notice how the patient reacted?*

*Scaffolding* involved supporting the care workers by being present during the conversation, keeping in the background as much as possible. As the care workers became more competent the team members would withdraw *(fade)* the support *(scaffolding)* and transfer the responsibility to the care workers: “*Many just need a “little push” and encouragement to talk with the patient alone”* As *the mobile teaching team* saw that the care workers increased their competency, they used *articulation, reflection and exploration*, by initiating dialogues to help the care workers to reflect and explore how they handled the situation.

Although developing individual care workers’ spiritual and existential care competency is important, the quality of spiritual and existential care also rests on other variables, such as the general work place culture, philosophy, leadership and organization of care [77]. Henceforth, *the mobile teaching team’s* descriptions of individual care workers’ competency improvement does not provide a picture of the overall impact of their situated bedside teaching in the nursing homes and home care settings [26].

A less qualified workforce increasingly dominates nursing homes and homecare nursing at a time of increasing prevalence of complex heath concerns [59, 78, 79]. Our results suggest that developing mobile expert nurse teaching teams in other relevant fields of nursing (such as hospice, dementia and geriatric care) may be a pedagogically effective and practical means to redress the widening gap between work force quality and the demand for care in primary health care [78]. Compared to the expenses of arranging education sessions and releasing staff to attend them, workplace learning under the guidance of mobile expert nurses may be a time and cost efficient means to improve the quality of nursing care in primary healthcare. Henceforth, Nicole and Reid [80] recommend employers to consider educational approaches that encourage workplace learning.

## Conclusions

Situated bedside teaching provided by expert mobile hospice nurses may be an efficient way to develop care workers’ courage and competency to provide spiritual and existential end-of-life-care. Further research is recommended on the use of mobile expert nurse teaching teams to improve nursing competency in the primary health care sector.
